# Multiple routes to territory inheritance in Florida Scrub‐Jays (*Aphelocoma coerulescens*)

**DOI:** 10.1111/1365-2656.70268

**Published:** 2026-05-26

**Authors:** Sarah K. Beres, Tori D. Bakley, Jeremy Summers, John W. Fitzpatrick, Sahas Barve

**Affiliations:** ^1^ University of Nebraska Lincoln Lincoln Nebraska USA; ^2^ Archbold Biological Station Venus Florida USA; ^3^ University of Rochester Rochester New York USA; ^4^ Cornell Lab of Ornithology Ithaca New York USA

**Keywords:** cooperative breeding, dispersal, gender dynamics, territory acquisition, territory inheritance

## Abstract

1. In many animals, being the dominant owner of a territory is a pre‐requisite for reproductive success. Acquisition of territory by inheritance, where at least a portion of the new (breeding) territory is acquired from the natal territory, characterizes many social animals and offers multiple benefits over dispersing away from natal ground. Territory dynamics change rapidly and are difficult to objectively quantify. Additionally, little is known regarding the non‐dispersive sex's potential to inherit.
2. We spatially analysed decades of territory maps to identify all nontrivial instances of natal territory inheritance in first‐time breeding Florida Scrub‐Jays (*Aphelocoma coerulescens*) and compared initial reproductive success of those that gained breeding status through any form of inheritance versus by other means. Among inheritors, we studied how first‐time territory acquisition strategies differed between the sexes.
3. Between 1982 and 2024, 139 out of 1014 first‐time breeders gained significant natal territory through inheriting the entire natal territory upon the death of both parents, pairing with an adjacent, widowed neighbour and incorporating some natal ground, or pairing with a novice disperser and budding off of their natal territory (or some combination of two).
4. Among all breeders that inherited substantial portions of natal territory, males did so about twice as often as females (97 vs. 42 instances, respectively). Birds that inherited natal territory did not have higher initial reproductive success than those that did not inherit. Comparing reproduction among birds who inherited, males and females did not differ either. Proportions of territory gained from the natal territory differed markedly between the sexes. Males gained a significantly higher percentage of their natal territory than females, and inherited territory made up a greater portion of their new territories. Males and females took different routes to inheritance: females showed higher plasticity in their acquisition strategy and more frequently paired with neighbouring, experienced widows on large territories, while males more commonly established new, relatively small territories with dispersing, novice females.
5. Our study demonstrates the value in objectively quantifying field‐gathered spatial data, as it revealed a previously unidentified trend of opportunistic female dispersals with beneficial fitness consequences.

## Graphical abstract

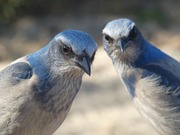

## 1 INTRODUCTION

Acquiring and maintaining a territory is a crucial step towards gaining individual fitness for a large proportion of the world's vertebrates, especially among birds (Brown, 1969; Howard, 1920; Nice, 1941). High‐quality territories are a limited resource and costly to acquire and defend, but provide prey, adequate nesting sites, shelter from predation and insurance in case of stochastic changes to habitat (Brown, 1969; Fitzpatrick & Bowman, 2016; Stallcup & Woolfenden, 1978; Wiley, 1973). Studying changes in territory dynamics in wild populations is challenging, as territory acquisition by new breeders may happen in a few minutes (Krebs, 1971), take place over a week (Barve et al., 2020) or even longer (Woolfenden & Fitzpatrick, 1984), and the exact boundaries of territories are often hard to define across the year (Fedy & Stutchbury, 2005; Maher & Lott, 1995). Additionally, most natal dispersal in birds occurs too early in life or over too great a geographic area in one or both sexes to evaluate easily.

Cooperative breeders often exhibit strong philopatry and short dispersal distances, allowing for the study of dispersal tradeoffs among individuals. In many cooperatively breeding taxa, sexually mature offspring delay their dispersal and breeding, remaining at their natal territory for up to several years (Emlen, 1982). These ‘helpers’ may improve their individual fitness by taking advantage of reliable natal resources in the face of ecological constraints or improve inclusive fitness by enhancing the survival of younger siblings, or both (Cockburn, 1998; Emlen, 1982; Hamilton, 1963; Johnson et al., 2023). In addition, a significant benefit of delaying dispersal is that helpers often gain breeding positions through inheritance (Funston et al., 2003; Koenig et al., 2023; Komdeur & Edelaar, 2001b; Woolfenden & Fitzpatrick, 1978). Acquiring a territory via inheritance may have significant energetic and fitness advantages over breeding farther away from the natal territory. Familiarity with local habitat and neighbouring territory holders may facilitate increased survival and/or elevated reproductive success among inheritors, although the specific mechanism underlying these benefits remains unclear (Fitzpatrick & Bowman, 2016; Komdeur & Edelaar, 2001b; Randall, 1989; Suh et al., 2020). Beyond this, recent studies hypothesize that family lineages may acrue ‘intergeneration wealth’ when a resource is passed from parent to offspring, suggesting that family lineages lacking such advantages face genetic costs over generations (Holekamp et al., 2012; Smith et al., 2022; Watson et al., 1994).

Territory inheritance in cooperatively breeding species can occur by various mechanisms depending on the mating system. In some polygynandrous species, individuals can gain natal territory by becoming a co‐breeder alongside their parents or by replacing them following turnover (Barve et al., 2019; Mumme et al., 1983). In systems with high rates of extra‐pair parentage, helpers may even inherit the breeding position of a deceased, same‐sex parent and form a social pairing with their opposite‐sex parent (Cockburn et al., 2008; Koenig & Dickinson, 2016; Webster et al., 2004). In monogamous species, helpers may inherit a natal breeding position following the death or disappearance of their parents (Cockburn, 1998). Alternately, many gain natal territory through a process known as budding (Komdeur & Edelaar, 2001b; Woolfenden & Fitzpatrick, 1978). As opposed to complete inheritance, territory budding refers to the formation of a territory bordering the natal territory, where the new territory retains significant natal area. Budding is accompanied by defence against the natal group and pairing with a mate not previously a member of the natal group (Komdeur & Edelaar, 2001b; Nichols et al., 2012; Woolfenden & Fitzpatrick, 1978).

In many species, helping behaviours have been linked to the potential for inheriting a natal breeding position, which is underscored by greater helper rates in the philopatric sex (Capilla‐Lasheras et al., 2024; Dickinson et al., 2016; Koenig et al., 2023; Komdeur & Edelaar, 2001b; Thorley et al., 2023; Woolfenden & Fitzpatrick, 1978). In meerkats (*Suricata suricatta*), for example, females are the philopatric sex and take on a significant role in raising non‐descendent young while male helpers disperse early, likely due to their inability to inherit (Clutton‐Brock et al., 2002). The dispersive sex, however, tends to disperse far and early, making them difficult to study and suggesting that fewer benefits exist to remaining at home. While territory inheritance is well studied in the philopatric sex (typically males for birds), it is not yet well documented whether and how the typically dispersive sex inherits natal ground and, if so, under what circumstances. Additionally, while many studies of cooperative breeders (including both birds and mammals) have examined inheritance in relation to body condition, natal territory investment, long term reproductive success, survival and variation in surrounding male breeding vacancies (Fitzpatrick & Bowman, 2016; Humphries et al., 2021; Kingma et al., 2017; Komdeur & Edelaar, 2001a; Legge & Cockburn, 2000; Lindström, 1986; Suh et al., 2020; Woolfenden & Fitzpatrick, 1978), we are aware of no studies that have quantified the *amount* of territory acquired from their natal territory by the new breeder, regardless of sex. Here we applied geo‐spatial analyses to measure spatial overlap of territories during sequential years before and after new‐pair formation to identify and quantify instances of territory inheritance in a long‐term study of Florida Scrub‐Jays (*Aphelocoma coerulescens*, FLSJ). Our aims were (1) to investigate if early reproductive benefits are associated with inheriting territory versus by other means, (2) to quantify the modes, relative frequency and area inherited by male and female helpers in their newly acquired territory and (3) to test whether the sexes differ in the circumstances by which they inherit natal territory.

## 2 MATERIALS AND METHODS

### 2.1 Study system

We analysed the long‐term demography dataset on Florida Scrub‐Jays collected at Archbold Biological Station (27° 10′ 52.35″ N 81° 21′ 9.11″ W) between 1982 and 2024, where at any given time ~ 98% of the FLSJs in the study population are colour‐banded for individual identification and the entire study population is censused monthly (Fitzpatrick & Bowman, 2016; Woolfenden & Fitzpatrick, 1984). The Florida Scrub‐Jay is a highly monogamous, cooperatively breeding bird endemic to Florida. FLSJs live in family groups with two dominant breeders and 0–4 offspring helpers of either sex (Fitzpatrick & Bowman, 2016). FLSJs reside in fire‐maintained, xeric scrub‐oak habitat, where they maintain large territories (~10 ha, Fitzpatrick & Bowman, 2016; Woolfenden & Fitzpatrick, 1984).

### 2.2 Territory mapping

Florida Scrub‐Jays live year‐round on strongly defended territories, most of which are tightly contiguous with one another along stable, extraordinarily narrow boundaries (Barve et al., 2025; Woolfenden & Fitzpatrick, 1984). Annually during the peak of breeding, we mapped all FLSJ territory boundaries in our study tract by documenting boundary disputes between family groups. For territories that abut unoccupied scrub or unsuitable habitat (e.g. pine or bayhead forest, open pasture, citrus groves) we noted as a boundary the perimeter of the occupants' regularly visited home range. All jays in our study tract have become habituated to humans, which we enhance by infrequently rewarding their close approach with small peanut morsels. This habituation has allowed us to document natural behaviours that are typically difficult to observe in detail, such as nesting activities and territory defence.

**FIGURE 1:**
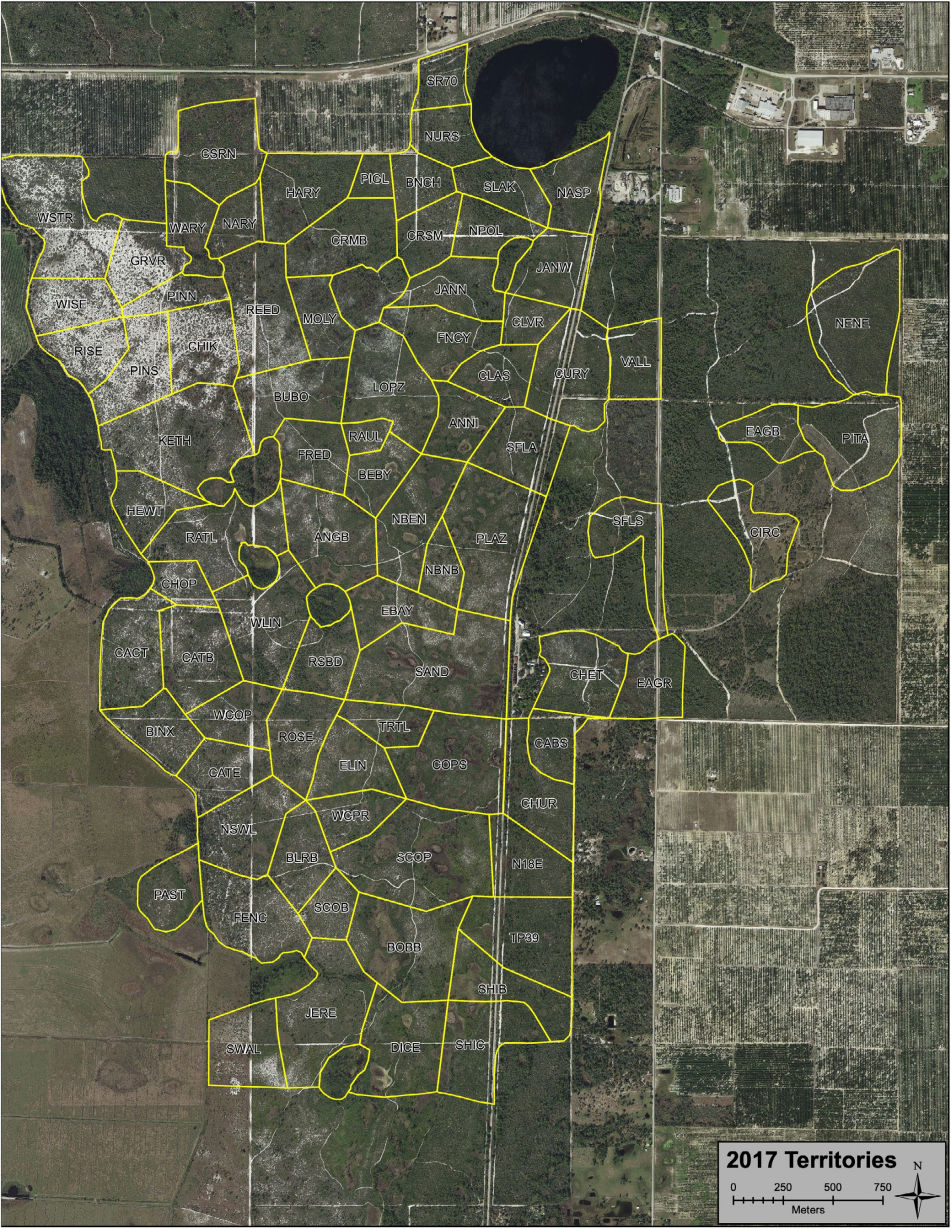
Territory maps for the Archbold Biological Station FLSJ demography tract in 2017. Yellow lines represent territory boundaries.

During the spring mapping period (early April through mid‐May) we visit all territories on multiple occasions to record territorial disputes between neighbours. Such disputes are regular occurrences in the daily lives of FLSJ family groups, especially during nesting season. Upon spotting one another, jays of both sexes in adjoining groups commence active territorial vocalizations and several kinds of ritualized displays, often flying long, undulating flights paralleling their shared boundary while calling repeatedly (see detailed behavioural descriptions in Woolfenden & Fitzpatrick, 1984). Territory mapping was completed between dawn and late morning before elevated temperatures and wind make the jays less active. As we encounter jay groups, we gradually coax them towards their boundaries by capitalizing on their general attraction to us. As soon as they spot one another, their attention shifts to territorial calling and active displays at the shared territory boundary. In the field, we digitally log all such points of dispute via personal mobile devices using the ‘Field Maps’ app (ESRI, 2024). Locations where three family groups engage in territory squabbles are especially informative, as they help define the emerging ‘honeycomb’ of contiguous territories (see Figure 1 for an example). The resulting georeferenced ArcInfo database (ESRI, ArcGIS 9.2) permits direct overlay comparisons between years, as performed for this study. Repeated visits to suspected or previously documented boundaries routinely confirm that their precise locations are recognized and conspicuously respected by the jays, remaining stable over months, years and in some cases, even decades.

### 2.3 Demography and social classification

To isolate novice breeding attempts and categorize mates, we first used monthly census data to track group membership and composition for each focal FLSJ across their lives. We measured reproductive success of novice breeders in their first breeding year as the number of offspring successfully fledged (surviving until day 18 post‐hatch).

**FIGURE 2:**
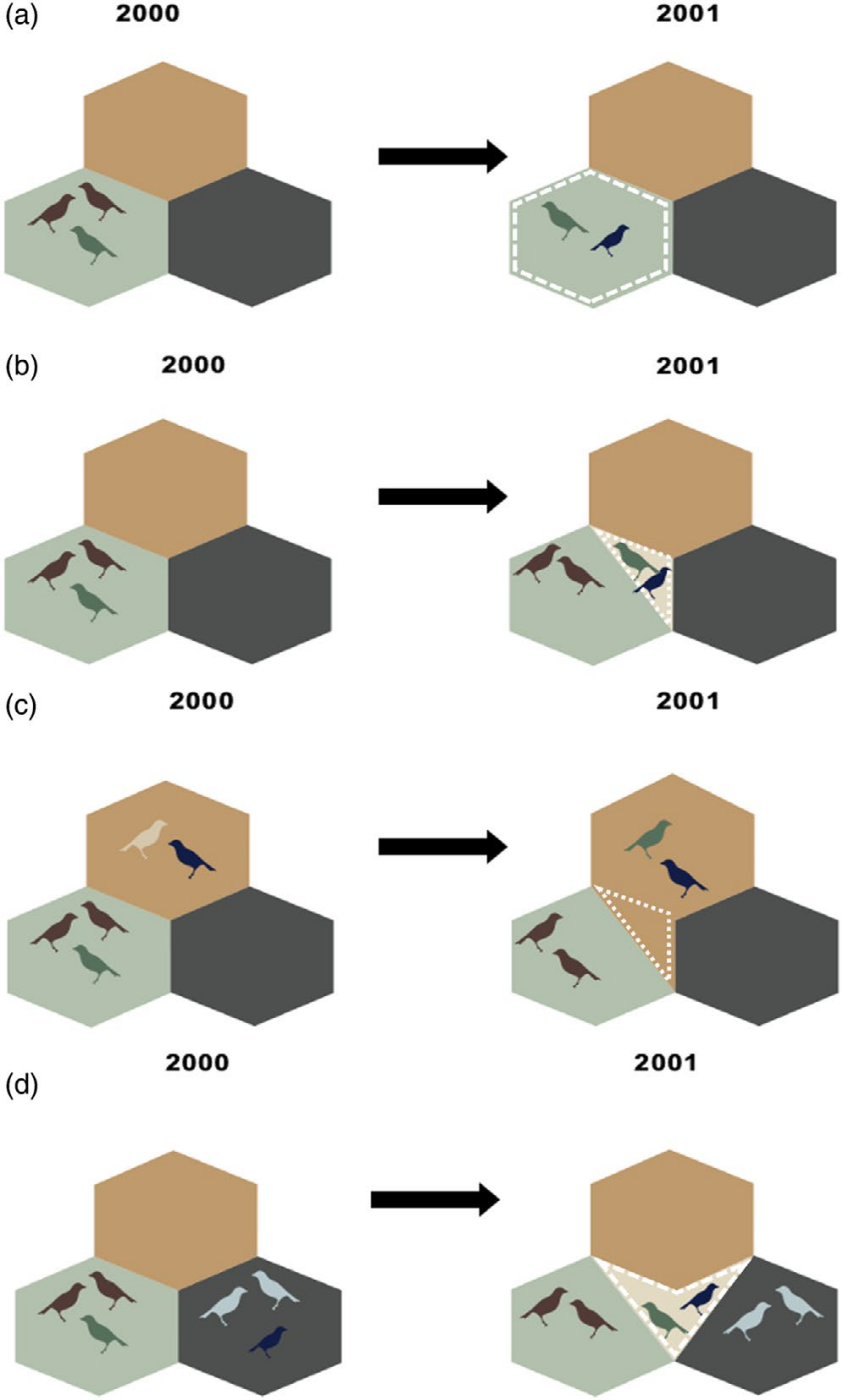
Graphic representations of various routes to territory inheritance in FLSJs. Spatial overlap between the natal territory in year 2000 and the territory formed by the new pair in year 2001 is outlined in a white, dashed line. (a) Succession (complete inheritance), the helper (green) gains natal territory following the death or disappearance of their parents (brown) and mates with another jay (dark blue). (b) Budding, the helper (green) pairs with a novice jay (dark blue) from a non‐adjacent territory and buds off their own, new territory. This usually follows natal territory expansion and subsequent defence by the helper against their own natal group. (c) Bud‐replacement, the helper (green) fills a breeding vacancy left by the death of a breeder (tan) in the adjacent territory, pairs with the widow (blue) and assumes ownership of a portion of the natal territory. (d) Joint‐budding, two neighbouring helpers (green and blue) bud simultaneously, both inheriting portions of their natal territory.

FLSJs commonly acquire territories to become breeders via several non‐mutually exclusive ways: (1) mate replacement (most common): an individual replaces a dead or disappeared mate on the widow's pre‐existing territory; (2) de novo (relatively rare), where a pair of jays carves out a new territory amidst an existing network of territories or unoccupied habitat away from either jay's natal territory; (3) inheriting their entire natal territory after both parents had died or vacated the natal territory (this will be referred to as ‘succession’ going forward; see Figure 2a) or (4) through territory budding, in which a new territory is formed by a helper adjacent to their parent(s), partially retaining some amount of natal territory. In budding, a helper actively aids in the expansion of the natal territory in the year prior to budding and subsequently defends against neighbours and the natal group itself (Figure 2b). To gain competitive breeding positions and territory, FLSJs often combine multiple methods (e.g. Figure 2c,d) to maximize gains and take advantage of current events. Bud‐replacement occurrs when a jay mates with a widowed neighbour and the subsequent territory includes a portion of the focal jay's natal territory (Figure 2c) and joint‐budding occurrs when two novice, neighbouring opposite‐sex helpers pair with each other, bud simultaneously and the new territory includes portions of both natal territories (Figure 2d).

To further evaluate the different ways in which a FLSJ might inherit natal territory, all nontrivial instances of inheritance were categorized based on mate type and status of parents (i.e. whether the natal territory was still active). Mates of new breeders were divided into four types based on their dispersal strategy and breeding experience: novice dispersers, novice residents, experienced residents and experienced dispersers. Novice dispersers were assumed to have no prior breeding experience and dispersed from a non‐adjoining territory (many of these were new immigrants into the study tract, typically aged as hatch‐year using moult criteria; Pyle, 1997). Mates described as novice residents were known to have no breeding experience and inherited portions of their own territory in the same year as the breeder in question's first attempt (see Figure 2d).

Overwhelmingly, most cases of territory inheritances thus far identified in FLSJs have been by males, with female‐budding occurring only rarely (Woolfenden & Fitzpatrick, 1978). Female helpers disperse earlier and farther than males, and hence are less likely to bud or replace a close‐by neighbour (Suh et al., 2020). Older male FLSJs provision nestlings and engage in territory defence more often than female helpers of the same age (Mumme, 1992). This increased investment by male helpers is hypothesized to contribute towards beneficial territory acquisition opportunities such as budding or inheritance (Fitzpatrick & Bowman, 2016; Woolfenden & Fitzpatrick, 1977, 1984).

### 2.4 Quantifying spatial composition of territories

We identified instances of inheritance using the *st_area*() and *st_intersection*() functions in package *sf* (Pebesma et al., 2023). For all territories in which one or both FLSJs were breeding for the first time, territory polygons were tested for overlap with the new breeder's natal territory from the year prior to breeding. If an overlap existed, we calculated the total area of the newly acquired territory and the proportion of overlap between it and both the natal territory and the new territory. Instances where more than 5% of the natal territory was gained were treated as inheritance and included in subsequent analyses.

### 2.5 Statistical analyses

All statistical analyses were done in R (version 4.2.3; R Core Team, 2023). We used generalized Poisson mixed‐effect models with a zero‐inflated component (ZIPG), zero‐inflated Poisson mixed‐effect (ZIP), Gaussian generalized linear mixed‐effect models, beta generalized linear mixed‐effects models and *χ* ^2^ tests to test our hypotheses (see Table 1 for details of each model) using historic territory name (unique identifiers kept specific to the general area within the study tract over the generations), historic natal territory name and year of acquisition as random effects in the mixed models when appropriate. All models were constructed using the package *glmmTMB* (Brooks et al., 2017).

**TABLE 1:** Outline of Hypotheses (GLMM, generalized linear mixed‐effect model; ZIP, Zero‐inflated Poisson mixed‐effect model; ZIPG, Generalized Poisson mixed‐effect model with a zero‐inflation component).

Prediction	Rationale	Dep. variable	Fixed effects	Aim and analysis	References
First‐year breeding success will not differ between breeders that do vs. do not inherit significant portions of natal territory	Territory budding, which makes up a high proportion of these inheritances, is a lengthy and energetically costly process that will equalize the benefits of territory inheritance	Number of fledglings produced	Sex, mate type, territory area (m^2^), inheritance (y/n), age (year)	Aim 1: Model 1 (*ZIPG*)	Fitzpatrick and Bowman (2016), Suh et al. (2020) and Tringali et al. (2020)
Inheriting females will see higher initial reproductive success than males	Inheriting females will more often have mated with experienced neighbours on pre‐established territory, allowing them to allocate more energy towards reproduction	Number of fledglings produced	Sex of inheriting breeder (M/F), mate type, territory area (m^2^) and age (year)	Aim 2: Model 2 (*ZIP*)	Kingma et al. (2017)
Inherited territories will be smaller for males than females	Males will more often bud, having to defend from both the natal territory and all other neighbours, resulting in a smaller territory	Area of ‘new’ territory (m^2^)	Sex of inheriting breeder (M/F), area of the natal territory (m^2^)	Aim 2: Model 3 (*Gaussian GLMM*)	Fitzpatrick and Bowman (2016) and Mumme (1992)
Males will gain more of their natal territories' area than females	Males more actively help their parents grow their territory, a part of which they may inherit	Area overlap / area natal territory (%)	Sex of inheriting breeder (M/F), area of the natal territory (m^2^)	Aim 2: Model 4 (*beta GLMM*)	Greenwood (1980) and Suh et al. (2020)
Inheriting females will gain a higher proportion of area from neighbouring territories than from her natal territory	Females are more likely to inherit natal area upon replacing a neighbouring widow, area which supplements an already established territory's	Area overlap / area ‘new’ territory (%)	Sex of inheriting breeder (M/F), area of the natal territory (m^2^) (*Model*: *beta GLMM*)	Aim 3 Model 5 (*beta GLMM*)	Greenwood (1980) and Suh et al. (2020)
Mate preference will differ significantly between inheriting males and females	Females disperse earlier and further than males, implying that inheriting males will more often mate with novice, dispersing females and inheriting females will more often mate opportunistically with established neighbour males	Mate type	Sex of inheriting breeder (M/F)	Aim 3: Table 3 (*χ* ^2^ test)	Fitzpatrick and Bowman (2016), Suh et al. (2020) and Tringali et al. (2020)

#### 2.5.1 Aim 1

We first analysed initial reproductive success (number of young successfully fledged) using ZIPGs and ZIPs (Table 2: models 1–2). We analysed the difference in reproductive success between individuals who inherited significant portions of their natal territory (>5%) and those who did not, controlling for sex, mate type, territory size (m^2^) and age (years). We then tested for differences in first‐year reproductive success among inheritances depending on sex, mate type, territory size (m^2^) and age (years).

Diagnostic testing using the *testZeroInflation*(), *testOverdispersion*() and *testResiduals*() functions from package *dHARMA* (Hartig, 2024) revealed that standard Poisson models were underdispersed and failed to account for the high frequency of birds who failed to fledge any young in their first breeding year. To address this, we compared Poisson models with a constant zero‐inflation component (ziformula = ~1) and one related to fixed effects (ziformula = ~ fixed effects), with the latter providing better fit (AIC >2) in Model 1. Model 2 failed to converge for a generalized Poisson distribution, suggesting better representation by the ZIP distribution rather than ZIPG. See Table S1 for more details on model selection.

#### 2.5.2 Aim 2

For model 3 (Table 2), we used a gaussian GLMM to test whether territory sizes (m^2^) differed between males and females that inherited substantial portions of their natal territory (>5%), excluding cases of succession (i.e. the natal territory still existed: budding, bud‐replacement and joint‐budding). A gaussian GLMM was the most appropriate for continuous measures of data such as area. Natal territory area was added as a fixed effect to control for variation in natal territory size.

Models 4–5 (Table 2) evaluated proportional data using beta regression, which is appropriate for data bounded between 0 and 1. Proportions equal to 1 were transformed to 0.999 prior to analysis. Model 4 tested for differences between the sexes in percentage of the natal territory that the helper retained after acquiring the new territory (i.e. area overlap in relation to the entire natal territory). Next, we compared sex and the % natal territory makeup of the new territory to analyse the source of area for these new territories (i.e. area overlap in relation to the new territory). To control for variation in natal territory quality, area of the natal territory was added as a fixed effect in models 4–5.

For all models described, we assessed model fit using R package *DHARMa* (Hartig, 2024). We checked for overdispersion, evaluated residual patterns with simulated residual plots using the *testResiduals*() function and checked for significant collinearity using the *checkcollinearlity*() function from package *performance* (Lüdecke et al., 2025). Table S1 outlines all relevant information for intermediate models. See Table S1 in supplemental material for summaries of intermediate models.

#### 2.5.3 Aim 3

Finally, we used *χ* ^2^ analyses with the *chisq.test*() function from base R (R Core Team, 2023)—appropriate for comparing the distributions of categorical data—to test whether new male and female breeders differed in their mate choice based on mate dispersal and breeding experience, as well as in their overall strategies for inheriting natal territory.

## 3 RESULTS

We analysed territory acquisition by 1014 FLSJ new breeders (516 females and 498 males) and identified 139 instances in which a new breeder inherited a significant portion (>5%) of their natal territory. Of these, 42 were females (30%) and 97 were males (70%). In 56 of these instances, breeders acquired territory via succession, involving 24 females (43%) and 32 males (57%). A total of 83 instances of non‐succession inheritance occurred (i.e. budding, bud‐replacement and joint‐budding), 18 of which were females (22%) and 65 of which were males (78%). In 78 additional cases of territories that gained between 0% and 5% of their natal territory, 19 were by females (24%) and 59 by males (76%).

### 3.1 Aim 1

First‐time breeders who inherited portions of natal territory did not have significantly higher first‐year reproductive success compared to those that did not inherit. Sex, area of the new territory and mate type also were not significantly different (Table 2: Model 1) in both the conditional and zero‐inflated models. Our model describes only age as a negative predictor for zero‐inflation (Table 2: Model 1). First‐year reproductive success of inheriting males and females did not differ significantly, regardless of mate type or area of the new territory (Table 2: Model 2).

**TABLE 2:** Results for all final models.

Model 1: ZIPG, Total # fledged ~ Sex + Mate Type + Inheritance + Area New Territory + Age + (1|TerritoryID) + (1|NatalTerritoryID) + (1|year)				
Fixed effects	Estimate	Std. error	*z*‐value	Pr(>|*t*|)
Conditional				
Intercept	0.885	0.06	15.90	**<2e‐16**
Sex	0.002	0.03	0.06	0.950
Mate type (ExpRes)	0.063	0.04	1.48	0.139
Mate type (NovDisp)	0.001	0.05	0.019	0.984
Mate type (NovRes)	0.030	0.06	−0.49	0.623
Inheritance (Y)	−0.014	0.04	−0.35	0.727
Area of territory	0.016	0.02	0.89	0.375
Age	0.001	0.01	0.07	0.941
Zero‐inflation				
Intercept	0.624	0.27	2.36	**0.019**
Sex	−0.072	0.13	−0.54	0.592
Mate type (ExpRes)	−0.106	0.20	−0.52	0.605
Mate type (NovDisp)	0.331	0.20	1.67	0.095
Mate type (NovRes)	−0.065	0.27	−0.24	0.812
Inheritance (Y)	−0.244	0.19	−1.27	0.204
Area of territory	−0.081	0.07	−1.20	0.232
Age	−0.286	0.07	−3.90	**9.47e‐05**

Model 2: ZIP, Total # fledged ~ Sex + Mate Type + Area New Territory + Age + (1|TerritoryID) + (1|NatalTerritoryID) + (1|Year)				
Fixed effects	Estimate	Std. Error	*z*‐value	Pr(>|*t*|)
Intercept	0.597	0.35	1.71	0.088
Sex (Male)	−0.129	0.19	−0.67	0.506
Mate type (ExpRes)	6.89e‐05	0.25	0.000	1.000
Mate type (NovDisp)	0.237	0.25	0.95	0.341
Mate type (NovRes)	0.092	0.39	0.32	0.753
Area of territory	0.152	0.09	1.74	0.082
Age	0.055	0.08	0.67	0.501

Model 3: Gaussian GLMM, Area Territory ~ Sex + Area New Territory + (1|TerritoryID) + (1|NatalTerritoryID) + (1|Year)				
Fixed effects	Estimate	Std. error	*z*‐value	Pr(>|*t*|)
Intercept	0.485	0.22	2.17	**0.030**
Sex (Male)	−0.568	0.24	−2.42	**0.016**
Area of natal territory	0.243	0.10	2.43	**0.015**

Model 4: Beta GLMM, (Area Overlap/Natal Area) ~ Sex + Area Territory + (1|TerritoryID) + (1|NatalTerritoryID) + (1|Year)				
Fixed effects	Estimate	Std. Error	*z*‐value	Pr(>|*t*|)
Intercept	−2.068	0.27	−7.72	**1.19e‐14**
Sex (Male)	0.483	0.24	1.99	**0.047**
Area of natal territory	−0.014	0.10	−0.15	0.880

Model 5: Beta GLMM, (Area Overlap/Territory Area) ~ Sex + Area Territory + (1|TerritoryID) + (1|NatalTerritoryID) + (1|Year)				
Fixed effects	Estimate	Std. Error	*z*‐value	Pr(>|*t*|)
Intercept	−0.979	0.33	−3.01	**0.003**
Sex (Male)	0.722	0.32	2.23	**0.025**
Area of natal territory	0.327	0.13	2.55	**0.011**

*Note*: Model 1 is a zero‐inflated generalized Poisson mixed‐effect model evaluating all first‐time breeding attempts between 1982 and 2024 (*n* = 1014). Inheritance was binomial and determined by whether breeders had gained substantial (>5%) amounts of their natal territory. Model 2 is a zero‐inflated Poisson model evaluating the reproductive success between those that inherited portions of natal territory (*n* = 139). Models 3–5 evaluated territories of individuals who gained substantial portions of their natal territory (>5%), excluding successions (*n* = 83). Mate type was categorized based on prior breeding experience (Exp = experienced, Nov = novice) and whether they had dispersed (Disp) or previously resided (Res) at the territory. Bolded values denote significant at *p* < 0.05.

### 3.2 Aim 2

**FIGURE 3:**
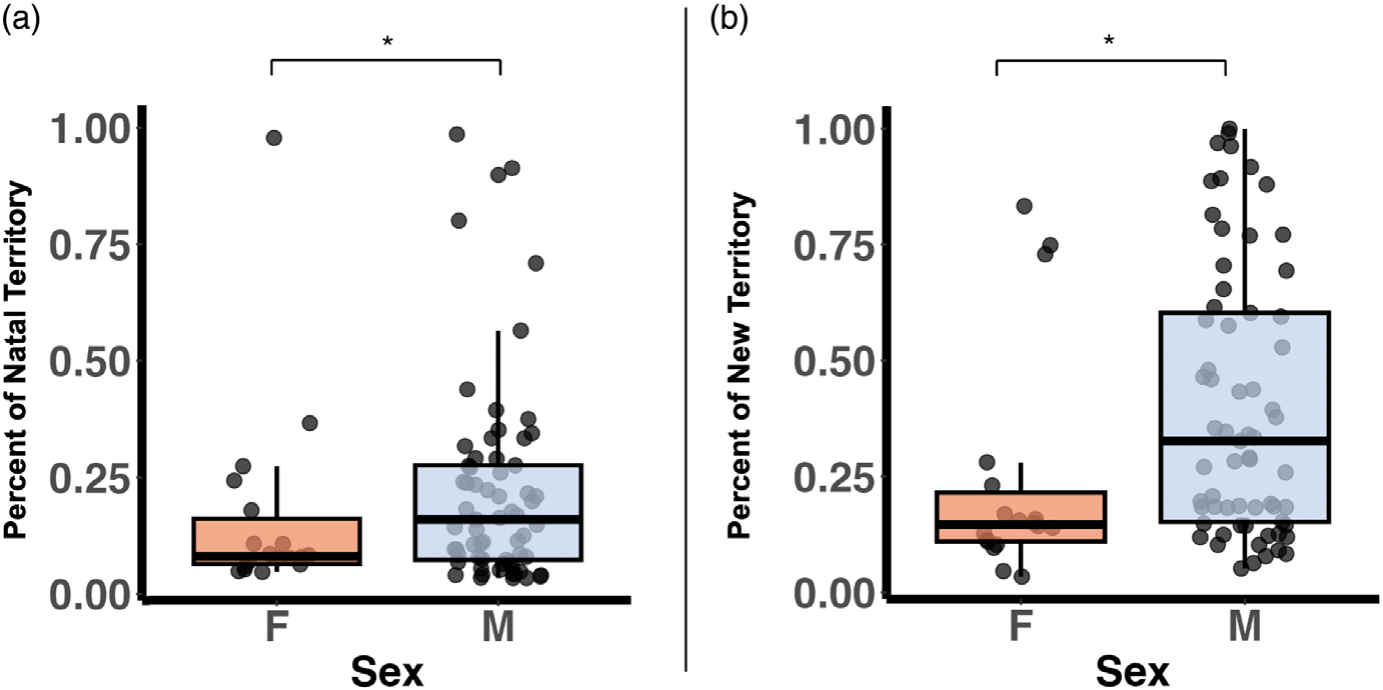
Area gained from the previous year's natal territory as a proportion of the natal territory (a) and the new territory (b) by 18 female and 65 male breeders who inherited substantial (>5%) portions of natal territory. Instances of succession were not included. Asterisks denote significant differences between sexes at an *α* level of 0.05.

Among territory inheritances which occurred while the natal territory was still active (i.e. budding, bud‐replacement and joint‐budding), the size of females' new territories was significantly greater than that of males (Table 2: Model 3). Males gained a significantly higher proportion of their natal territory than females (Table 2: Model 4; Figure 3a), and natal territory made up a greater percentage of their newly occupied territory (Table 2: Model 5; Figure 3b). Area of the natal territory in the year prior to first breeding was a significant positive predictor for the area of the inherited territory and the proportion of the new territory made up by inherited area (Table 2: Model 3 and 5).

### 3.3 Aim 3

*χ* ^2^ analyses revealed significant differences in the distribution of categorical mate type (Table 3) and inheritance strategies (Table 4) of males and females. Females mated most frequently with experienced neighbours (36% of all female inheritances) and males most frequently with novice, dispersing females (49% of all male inheritances). Inheriting males were most likely to do so by budding (43% of all male inheritances) while females were most likely to do so by succession (21% of all female inheritances), though their inheritance strategies were more evenly distributed across all strategies.

**TABLE 3:** Proportion and count of natal territory inheritances based on dispersal strategy and breeding experience of the mate to the focal new breeder (*χ* ^2^ = 12.116, df = 3, *p* = 0.007; *n* = 139).

Sex	Mate category	Count	Prop
*M* (*n* = 97)	Novice Disperser	48	0.49
	Novice Neighbour	8	0.08
	Experienced Disperser	19	0.20
	Experienced Neighbour	22	0.23
*F* (*n* = 42)	Novice Disperser	10	0.24
	Novice Neighbour	10	0.24
	Experienced Disperser	7	0.17
	Experienced Neighbour	15	0.36

**TABLE 4:** Proportion and count of natal territory inheritances based on inferred strategy to gain natal territory (*n* = 139, *χ* ^2^ = 22.283, df = 5, *p* < 0.001).

Sex	Strategy	Count	Prop
*M* (*n* = 97)	Succession × Neighbour Replacement	6	0.06
	Succession × Neighbour Inheritance	1	0.01
	Succession alone	25	0.26
	Bud‐replacement	16	0.16
	Joint‐bud	7	0.07
	Bud	42	0.43
*F* (*n* = 42)	Succession × Neighbour Replacement	8	0.19
	Succession × Neighbour Inheritance	7	0.17
	Succession alone	9	0.21
	Bud‐replacement	7	0.17
	Joint‐bud	3	0.07
	Bud	8	0.19

*Note*: ‘Succession’ necessitates the death or disappearance of both parents; ‘Succession × Neighbour Replacement’ implies the focal breeder additionally mated with a widowed neighbouring jay, while ‘Succession × Neighbour Inheritance’ implies they additionally mated with a novice neighbouring helper who simultaneously inherited. ‘Bud‐replacement’ implies that a helper filled a neighbouring breeding vacancy and subsequently inherited territory while the natal territory was still active. ‘Joint‐bud’ implies two novice, neighbouring helpers bud simultaneously where ‘Bud’ implies a helper attracted a novice, dispersing mate—both of which having occurred while the natal territory was still active. See Figure 2 for more details.

## 4 DISCUSSION

For cooperative‐breeding birds a prevailing observation has been that males are more likely than females to either entirely or partly inherit their natal territory, as they are more philopatric and typically help the dominant breeders more than do female helpers (Kingma, 2017; Koenig et al., 2023). Our results support elements of this generalization for the Florida Scrub‐Jay but also highlight that inheriting portions of the natal territory is not restricted to the philopatric sex. A nontrivial proportion of females that became breeders in our study site (42 of 516 or 8.1%) did inherit portions of their natal territory in the process (compared to 97 of 498 or 19% for male new breeders).

We found no first‐year reproductive benefit to territory inheritance, as individuals who inherited natal territory did not have higher initial reproductive success compared to those that gained territory by other means (e.g. replacement after dispersing; Table 2: Model 1). This is consistent with other findings for this species (Fitzpatrick & Bowman, 2016; Suh et al., 2020; Tringali et al., 2020). We expected females who inherited natal territory to have higher initial reproductive success since they are more likely to mate with experienced neighbours. Budding is a risky territory acquisition method, as budding males have lower initial survival and reproductive success compared to males that gain territory through other routes such as mate replacement (Fitzpatrick & Bowman, 2016; Komdeur & Edelaar, 2001b). However, among individuals that inherited substantial portions of natal territory, males and females did not differ in their initial reproductive success (Table 2: Model 2).

The sexes differed in three key aspects: (1) territory size, (2) source of the territory—specifically, the proportion of natal territory gained relative to both the natal and ‘new’ territories and (3) mate preference. Males were more likely to bud by inheriting greater proportions of natal territory and pairing with novice females. They did so by establishing relatively small territories largely consisting of area formerly in the natal territory. Females who inherited some natal ground more often became breeders on larger territories than males, typically by pairing with widowed resident males from adjacent neighbouring territories. As such, these territories had pre‐existing boundaries and included proportionally less natal territory than males (Tables 2 and 3; Figure 3).

Among individuals who inherited from active natal territories (i.e. budding, bud‐replacement and joint‐budding), birds inherited larger territories when their natal territories were large (Table 2, Model 3). This is important as territory size is positively associated with reproductive success in FLSJs (Fitzpatrick & Bowman, 2016). Males gained a significantly greater proportion of natal territory than females (Table 2: Model 4). Male helpers feed nestlings more frequently and remain at the natal territory for significantly longer than female helpers (Mumme, 1992; Suh et al., 2020). It is theorized that males compensate for this costly behaviour by gaining sought after breeding positions closer to home via budding or inheriting, which is supported by many similar studies and across several taxon (Barve et al., 2019; Cockburn, 1998; Freedberg & Wade, 2001; Hodge, 2007; Holekamp et al., 2012; Nichols et al., 2012; Woolfenden & Fitzpatrick, 1978). Perhaps as expected, given the diversity in cooperative‐breeding strategies, this tradeoff has many exceptions. For example, in Seychelles Warblers, helping behaviours have even been negatively correlated with an individual's ability to bud off their natal territory (Komdeur & Edelaar, 2001b). In communally breeding Banded Mongooses (*Mungos mungo*), both males and females are capable of inheritance, but males help significantly more while female helpers pursue earlier breeding opportunities (Hodge, 2007). FLSJs, on the other hand, are genetically monogamous with a single dominant pair per territory and have intense ties to all‐purpose territories, removing the potential for co‐breeding (Fitzpatrick & Bowman, 2016). In monogamous FLSJs, our results reveal that males inherit greater amounts of natal territory than females who gain breeding positions by the same means, suggesting a potential benefit to their greater energy expenditures while living in their natal territories.

Territory inheritance by females is more common in FLSJs than previously noted (Suh et al., 2020; Woolfenden & Fitzpatrick, 1978, 1984). Indeed, prior to the present analysis only six dispersal events in the entire long‐term database had been classified as instances of budding by females and, as of 1984, no female had been recognized as acquiring natal territory via bud‐replacement (Woolfenden & Fitzpatrick, 1984). Our objective, spatial approach to measuring territorial overlaps between successive years yielded 42 instances in which females gained breeding status while inheriting at least 5% of their natal territory. In contrast to male FLSJs, who most commonly formed relatively small territories by budding, females most often paired with neighbouring males, establishing on significantly larger territories than inheriting males. This suggests a substantial benefit for philopatric females, as large territories have been linked to several lifetime fitness advantages in FLSJs (e.g. reproductive success, adult and offspring survival; Fitzpatrick & Bowman, 2016; Mumme, 1992). Females likely gain this advantage by pairing more often with a neighbouring widow compared to males (41% vs. 31%, respectively, Table 3). Similar behaviour is seen in other cooperative breeders. In Purple‐backed Fairywrens (*Malurus assimilis*) and Superb Fairywrens (*Malurus cyaneus*), female helpers join non‐kin territories potentially to induce a resident male helper to bud (Cockburn et al., 2003; Johnson et al., 2023). Female fairy‐wrens thus take advantage of a helper male's establishment within an unrelated group to secure their own territory. In this regard, the dispersive sex appears to be more plastic in their acquisition of breeding positions. Generally, our findings suggest the need to reassess the frequency which the non‐philopatric sex inherits area or breeding positions among territorial species.

Males and females use different strategies to gain natal territory, with males often engaging in budding behaviour, while females are more opportunistic, taking advantage of stochastic breeding opportunities (i.e. natal territory succession following the death of parents and/or neighbouring breeding vacancies; Table 4) in the vicinity of their natal territory.

## 5 CONCLUSIONS

Our results confirm that female Florida Scrub‐Jays represent over a quarter of all cases in which first‐time breeders inherited portions of their natal territory. On average, the sexes differ in the strategies by which natal territory inheritance is accomplished. Males perform budding more frequently, typically attracting inexperienced, dispersing females and actively creating new, relatively small territories. Females more frequently acquire a portion of their natal ground by pairing with a neighbouring male, usually an experienced one. Our results underscore the importance of objectively measuring spatial relationships among territories between years, as doing so revealed previously under‐recognized levels of territorial inheritance by females. The prevalence of natal territory inheritance among females as well as males highlights that, among territorial cooperative breeders, significant benefits of philopatry can apply to both sexes. Even among the dispersive sex, plasticity in dispersal strategy allows individuals to capitalize on opportunities to become breeders without the considerable risks inherent in leaving home.

## AUTHOR CONTRIBUTIONS

Sarah K. Beres, Tori D. Bakley and Sahas Barve contributed the main question, idea and methodology. Sarah K. Beres and Jeremy Summer's analysed the data. John W. Fitzpatrick began the long‐term demography study and developed the methodology for territory mapping. Sarah K. Beres led the writing of the manuscript. All authors contributed critically to the drafts and gave final approval for publication.

## FUNDING INFORMATION

All research was funded by Archbold Biological Station.

## CONFLICT OF INTEREST STATEMENT

Authors have no conflicts of interest.

## STATEMENT ON INCLUSION

Our study analysed data collected as part of a long‐term demography study completed in rural Florida. Archbold Biological Station is home to researchers from all over the country and world, bridging together several different perspectives. Collaborations with several other establishments (within the local region, broader country and internationally) are a regular occurrence.

## Supporting information


**Table S1.** Dispersion, zero‐inflation and AIC scores for all intermediate and final models of Models 1–5 (Table 2). All conditional fixed effects remained constant (see Tables 1 and 2). Tests performed using the *TestZeroInflation*() and *TestOverdispersion*() functions from package *DHARMa* (Hartig, 2024). Bolded rows were final models presented.

## Data Availability

Data available from the Dryad Digital Repository https://datadryad.org/dataset/doi:10.5061/dryad.08kprr5gj (Beres et al., 2026).

## References

[jane70268-bib-0001] Barve, S. , Bakley, T. D. , Tringali, A. , Fitzpatrick, J. W. , & Bowman, R. (2025). Warm winters lead to increased reproductive effort, but lower reproductive success: Hidden costs of climate warming in a threatened bird. Ornithology, 142(1), ukae053. 10.1093/ornithology/ukae053

[jane70268-bib-0002] Barve, S. , Koenig, W. D. , Haydock, J. , & Walters, E. L. (2019). Habitat saturation results in joint‐nesting female coalitions in a social bird. The American Naturalist, 193(6), 830–840. 10.1086/703188 31094599

[jane70268-bib-0003] Barve, S. , Lahey, A. S. , Brunner, R. M. , Koenig, W. D. , & Walters, E. L. (2020). Tracking the warriors and spectators of acorn woodpecker wars. Current Biology, 30(17), R982–R983. 10.1016/j.cub.2020.07.073 32898494

[jane70268-bib-0004] Beres, S. K. , Bakley, T. D. , Summers, J. , Fitzpatrick, J. W. , & Barve, S. (2026). Data from: Multiple routes to territory inheritance in Florida Scrub‐Jays (*Aphelocoma coerulescens*). *Dryad Digital* Repository. https://datadryad.org/dataset/doi:10.5061/dryad.08kprr5gj 10.1111/1365-2656.70268 PMC13322170 42189840

[jane70268-bib-0005] Brooks, M. , Kristensen, K. , van Benthem, K. , Magnusson, A. , Berg, C. , Nielsen, A. , Skaug, H. , Maechler, M. , & Bolker, B. (2017). glmmTMB balances speed and flexibility among packages for zero‐inflated generalized linear mixed modeling. The R Journal, 9(2), 378–400. 10.32614/RJ-2017-066

[jane70268-bib-0006] Brown, J. L. (1969). Territorial behavior and population regulation in birds. The Wilson Bulletin, 81(3), 293–329. https://digitalcommons.usf.edu/wilson_bulletin/vol81/iss3/7

[jane70268-bib-0008] Capilla‐Lasheras, P. , Bircher, N. , Brown, A. M. , Harrison, X. , Reed, T. , York, J. E. , Cram, D. L. , Rutz, C. , Walker, L. , Naguib, M. , & Young, A. J. (2024). Evolution of sex differences in cooperation can be explained by trade‐offs with dispersal. PLoS Biology, 22(10), e3002859. 10.1371/journal.pbio.3002859 39446701 PMC11500963

[jane70268-bib-0009] Clutton‐Brock, T. H. , Russell, A. F. , Young, A. J. , Balmforth, Z. , & McIlrath, G. M. (2002). Evolution and development of sex differences in cooperative behavior in Meerkats. Science, 297(5579), 253–256. 10.1126/science.1071412 12114627

[jane70268-bib-0010] Cockburn, A. (1998). Evolution of helping behavior in cooperatively breeding birds. Annual Review of Ecology and Systematics, 29(1), 141–177. 10.1146/annurev.ecolsys.29.1.141

[jane70268-bib-0011] Cockburn, A. , Osmond, H. L. , Mulder, R. A. , Green, D. J. , & Double, M. C. (2003). Divorce, dispersal and incest avoidance in the cooperatively breeding superb fairy‐wren Malurus cyaneus. Journal of Animal Ecology, 72(2), 189–202. 10.1046/j.1365-2656.2003.00694.x

[jane70268-bib-0058] Cockburn, A. , Sims, R. A. , Osmond, H. L. , Green, D. J. , Double, M. C. , & Mulder, R. A. (2008). Can we measure the benefits of help in cooperatively breeding birds: the case of superb fairy‐wrens Malurus cyaneus? Journal of Animal Ecology, 77(3), 430–438. Portico. 10.1111/j.1365-2656.2007.01351.x 18312341

[jane70268-bib-0012] Dickinson, J. L. , Akçay, Ç. , Ferree, E. D. , & Stern, C. A. (2016). Western bluebirds: Lessons from a marginal cooperative breeder. In J. L. Dickinson & W. D. Koenig (Eds.), Cooperative breeding in vertebrates: Studies of ecology, evolution, and behavior (pp. 19–38). Cambridge University Press.

[jane70268-bib-0013] Emlen, S. T. (1982). The evolution of helping. I. An ecological constraints model. The American Naturalist, 119(1), 29–39. 10.1086/283888

[jane70268-bib-0059] ESRI . (2024). ArcGIS Desktop: Version 3.3. ESRI, Redlands, CA, USA.

[jane70268-bib-0014] Fedy, B. C. , & Stutchbury, B. J. M. (2005). Territory defence in tropical birds: Are females as aggressive as males? Behavioral Ecology and Sociobiology, 58(4), 414–422. 10.1007/s00265-005-0928-4

[jane70268-bib-0015] Fitzpatrick, J. W. , & Bowman, R. (2016). Florida scrub‐jays: Oversized territories and group defense in a fire‐maintained habitat. In J. L. Dickinson & W. D. Koenig (Eds.), Cooperative breeding in vertebrates: Studies of ecology, evolution, and behavior (pp. 77–96). Cambridge University Press.

[jane70268-bib-0016] Freedberg, S. , & Wade, M. J. (2001). Cultural inheritance as a mechanism for population sex‐ratio bias in reptiles. Evolution, 55(5), 1049–1055. 10.1111/j.0014-3820.2001.tb00621.x 11430641

[jane70268-bib-0017] Funston, P. J. , Mills, M. G. L. , Richardson, P. R. K. , & van Jaarsveld, A. S. (2003). Reduced dispersal and opportunistic territory acquisition in male lions (*Panthera leo*). Journal of Zoology, 259(2), 131–142. 10.1017/S0952836902003126

[jane70268-bib-0018] Greenwood, P. J. (1980). Mating systems, philopatry and dispersal in birds and mammals. Animal Behaviour, 28(4), 1140–1162. 10.1016/S0003-3472(80)80103-5

[jane70268-bib-0019] Hamilton, W. D. (1963). The evolution of altruistic behavior. The American Naturalist, 97(896), 354–356. 10.1086/497114

[jane70268-bib-0020] Hartig, F. (2024). *DHARMa: Residual diagnostics for hierarchical* (*multi‐level/mixed*) *regression models* (version 0.4.7) [R]. http://florianhartig.github.io/DHARMa/

[jane70268-bib-0021] Hodge, S. J. (2007). Counting the costs: The evolution of male‐biased care in the cooperatively breeding banded mongoose. Animal Behaviour, 74(4), 911–919. 10.1016/j.anbehav.2006.09.024

[jane70268-bib-0022] Holekamp, K. E. , Smith, J. E. , Strelioff, C. C. , Van Horn, R. C. , & Watts, H. E. (2012). Society, demography and genetic structure in the spotted hyena. Molecular Ecology, 21(3), 613–632. 10.1111/j.1365-294X.2011.05240.x 21880088

[jane70268-bib-0023] Howard, H. E. (1920). Territory in bird life. J. Murray.

[jane70268-bib-0024] Humphries, D. J. , Nelson‐Flower, M. J. , Bell, M. B. V. , Finch, F. M. , & Ridley, A. R. (2021). Kinship, dear enemies, and costly combat: The effects of relatedness on territorial overlap and aggression in a cooperative breeder. Ecology and Evolution, 11(23), 17031–17042. 10.1002/ece3.8342 34938490 PMC8668771

[jane70268-bib-0025] Johnson, A. E. , Welklin, J. F. , Hoppe, I. R. , & Shizuka, D. (2023). Ecogeography of group size suggests differences in drivers of sociality among cooperatively breeding fairywrens. Proceedings of the Royal Society B: Biological Sciences, 290(1995), 20222397. 10.1098/rspb.2022.2397 PMC10015324 36919434

[jane70268-bib-0026] Kingma, S. A. (2017). Direct benefits explain interspecific variation in helping behaviour among cooperatively breeding birds. Nature Communications, 8(1), 1094. 10.1038/s41467-017-01299-5 PMC5653647 29061969

[jane70268-bib-0027] Kingma, S. A. , Komdeur, J. , Burke, T. , & Richardson, D. S. (2017). Differential dispersal costs and sex‐biased dispersal distance in a cooperatively breeding bird. Behavioral Ecology, 28(4), 1113–1121. 10.1093/beheco/arx075

[jane70268-bib-0028] Koenig, W. D. , & Dickinson, J. L. (2016). Cooperative breeding in vertebrates. Cambridge University Press.

[jane70268-bib-0029] Koenig, W. D. , Haydock, J. , Dugdale, H. L. , & Walters, E. L. (2023). Territory inheritance and the evolution of cooperative breeding in the Acorn Woodpecker. Animal Behaviour, 205, 241–249. 10.1016/j.anbehav.2023.08.021

[jane70268-bib-0030] Komdeur, J. , & Edelaar, P. (2001a). Evidence that helping at the nest does not result in territory inheritance in the Seychelles warbler. Proceedings of the Royal Society of London B: Biological Sciences, 268(1480), 2007–2012. 10.1098/rspb.2001.1742 PMC1088842 11571047

[jane70268-bib-0031] Komdeur, J. , & Edelaar, P. (2001b). Male Seychelles warblers use territory budding to maximize lifetime fitness in a saturated environment. Behavioral Ecology, 12(6), 706–715. 10.1093/beheco/12.6.706

[jane70268-bib-0032] Krebs, J. R. (1971). Territory and breeding density in the Great Tit, *Parus major* L. Ecology, 52(1), 2–22. 10.2307/1934734

[jane70268-bib-0033] Legge, S. , & Cockburn, A. (2000). Social and mating system of cooperatively breeding laughing kookaburras (*Dacelo novaeguineae*). Behavioral Ecology and Sociobiology, 47(4), 220–229. 10.1007/s002650050659

[jane70268-bib-0035] Lindström, E. (1986). Territory inheritance and the evolution of group‐living in carnivores. Animal Behaviour, 34(6), 1825–1835. 10.1016/S0003-3472(86)80268-8

[jane70268-bib-0036] Lüdecke, D. , Makowski, D. , Ben‐Shachar, M. , Patil, I. , Waggoner, P. , Wiernik, B. , & Thériault, R. (2025). Performance: Assessment of regression models performance (version 0.13.0) [R]. n.

[jane70268-bib-0037] Maher, C. R. , & Lott, D. F. (1995). Definitions of territoriality used in the study of variation in vertebrate spacing systems. Animal Behaviour, 49(6), 1581–1597.

[jane70268-bib-0038] Mumme, R. L. (1992). Do helpers increase reproductive success? Behavioral Ecology and Sociobiology, 31(5), 319–328. 10.1007/BF00177772

[jane70268-bib-0039] Mumme, R. L. , Koenig, W. D. , & Pitelka, F. A. (1983). Reproductive competition in the communal Acorn Woodpecker: Sisters destroy each other's eggs. Nature, 306(5943), 583–584. 10.1038/306583a0

[jane70268-bib-0040] Nice, M. M. (1941). The role of territory in bird life. The American Midland Naturalist, 26(3), 441–487. 10.2307/2420732

[jane70268-bib-0041] Nichols, H. J. , Jordan, N. R. , Jamie, G. A. , Cant, M. A. , & Hoffman, J. I. (2012). Fine‐scale spatiotemporal patterns of genetic variation reflect budding dispersal coupled with strong natal philopatry in a cooperatively breeding mammal. Molecular Ecology, 21(21), 5348–5362. 10.1111/mec.12015 22994210

[jane70268-bib-0042] Pebesma, E. , Bivand, R. , Racine, E. , Sumner, M. , Cook, I. , Keitt, T. , Lovelace, R. , Wickham, H. , Ooms, J. , Müller, K. , Pedersen, T. L. , Baston, D. , & Dunnington, D. (2023). *sf: Simple features for R* (version 1.0–14) [R]. https://cran.r‐project.org/web/packages/sf/index.html

[jane70268-bib-0044] Pyle, P. (1997). Identification guide to north American birds: Columbidae to Ploceidae. Slate Creek Press.

[jane70268-bib-0045] R Core Team . (2023). R: A language and environment for statistical computing. R Core Team. https://www.R‐project.org/

[jane70268-bib-0046] Randall, J. A. (1989). Territorial‐defense interactions with neighbors and strangers in Banner‐tailed Kangaroo Rats. Journal of Mammalogy, 70(2), 308–315.

[jane70268-bib-0047] Smith, J. E. , Natterson‐Horowitz, B. , & Alfaro, M. E. (2022). The nature of privilege: Intergenerational wealth in animal societies. Behavioral Ecology, 33(1), 1–6. 10.1093/beheco/arab137

[jane70268-bib-0048] Stallcup, J. A. , & Woolfenden, G. E. (1978). Family status and contributions to breeding by Florida scrub jays. Animal Behaviour, 26, 1144–1156. 10.1016/0003-3472(78)90104-5

[jane70268-bib-0049] Suh, Y. H. , Pesendorfer, M. B. , Tringali, A. , Bowman, R. , & Fitzpatrick, J. W. (2020). Investigating social and environmental predictors of natal dispersal in a cooperative breeding bird. Behavioral Ecology, 31(3), 692–701. 10.1093/beheco/araa007

[jane70268-bib-0050] Thorley, J. , Bensch, H. M. , Finn, K. , Clutton‐Brock, T. , & Zöttl, M. (2023). Damaraland mole‐rats do not rely on helpers for reproduction or survival. Evolution Letters, 7(4), 203–215. 10.1093/evlett/qrad023 37475748 PMC10355180

[jane70268-bib-0051] Tringali, A. , Sherer, D. L. , Cosgrove, J. , & Bowman, R. (2020). Life history stage explains behavior in a social network before and during the early breeding season in a cooperatively breeding bird. PeerJ, 8, e8302. 10.7717/peerj.8302 32095315 PMC7020825

[jane70268-bib-0052] Watson, A. , Moss, R. , Parr, R. , Mountford, M. D. , & Rothery, P. (1994). Kin landownership, differential aggression between kin and non‐kin, and population fluctuations in red grouse. The Journal of Animal Ecology, 63(1), 39. 10.2307/5581

[jane70268-bib-0053] Webster, M. S. , Tarvin, K. A. , Tuttle, E. M. , & Pruett‐Jones, S. (2004). Reproductive promiscuity in the splendid fairy‐wren: Effects of group size and auxiliary reproduction. Behavioral Ecology, 15(6), 907–915. 10.1093/beheco/arh093

[jane70268-bib-0054] Wiley, R. H. (1973). Territoriality and non‐random mating in Sage Grouse, *Centrocercus urophasianus* . Animal Behaviour Monographs, 6, 85–169. 10.1016/0003-3472(73)90004-3

[jane70268-bib-0055] Woolfenden, G. E. , & Fitzpatrick, J. W. (1977). Dominance in the Florida Scrub Jay. The Condor, 79(1), 1. 10.2307/1367524

[jane70268-bib-0056] Woolfenden, G. E. , & Fitzpatrick, J. W. (1978). The inheritance of territory in group‐breeding birds. Bioscience, 28(2), 104–108. 10.2307/1307423

[jane70268-bib-0057] Woolfenden, G. E. , & Fitzpatrick, J. W. (1984). The Florida Scrub Jay: Demography of a cooperative‐breeding bird. Princeton University Press.

