# Supplementary material for: Multiple routes to territory inheritance in Florida Scrub‐Jays (Aphelocoma coerulescens)
Source: J Anim Ecol. 2026 May 26;95(7):1273–84. doi: 10.1111/1365-2656.70268 (PMC13322170; doi:10.1111/1365-2656.70268)
Supplement: Supplementary file 1 — Table S1. Dispersion, zero‐inflation and AIC scores for all intermediate and final models of Models 1–5 (Table 2). All conditional fixed effects remained constant (see Tables 1 and 2). Tests performed using the TestZeroInflation() and TestOverdispersion() functions from package DHARMa (Hartig, 2024). Bolded rows were final models presented. [file JANE-95-1273-s001.docx]

**Supplemental Materials -**

| Table S1: Dispersion, zero-inflation and AIC scores for all intermediate and final models of Models 1-5 (Table 2). All conditional fixed effects remained constant (see Tables 1-2). Tests performed using the *TestZeroInflation()* and *TestOverdispersion()* functions from package *DHARMa* (Hartig, 2024). Bolded rows were final models presented. | | | | | |
| --- | --- | --- | --- | --- | --- |
| **Name** | **Response Variable** | **Family** | **Zero-inflation Score** | **Dispersion Score** | **AIC** |
| Model 1b | First-time breeding attempts (n = 1014) | Poisson | ratioObsSim = 1.3071,  p <2.2e-16 | Dispersion = 1.0128,  p = 0.744 | 3232.191 |
| Model 1c | First-time breeding attempts (n = 1014) | Zero-inflated Poisson (zi = 1) | ratioObsSim = 1.0024,  p = 0.96 | Dispersion = 0.77649, p < 2.2e-16 | 2923.181 |
| Model 1d | First-time breeding attempts (n = 1014) | Generalized Poisson (zi = 1) | ratioObsSim = 1.0007,  p = 0.976 | Dispersion = 1.0128,  p = 0.744 | 2715.385 |
| **Model 1** | **First-time breeding attempts (n = 1014)** | **Zero-inflated, Generalized Poisson (zi = fixed effects)** | **ratioObsSim = 1.0011,**  **p = 0.968** | **Dispersion = 1.014,**  **p = 0.704** | **2694.013** |
| Model 2b | First time breeding attempts of inheritors (n = 139) | Poisson | ratioObsSim = 1.3095, p = 0.056 | Dispersion = 0.88438, p = 0.72 | 461.5995 |
| **Model 2** | **First time breeding attempts of inheritors (n = 139)** | **Zero-inflated Poisson (zi = 1) GLMM** | **ratioObsSim = 1.0022, p = 1** | **Dispersion = 0.80175, p = 0.12** | **435.9223** |
| **Model 3** | **Area of first territory (n = 83)** | **Gaussian GLMM** | **n/a** | **Dispersion = 1.0069, p = 0.904** | **-6.4** |
| **Model 4** | **% of natal territory (overlap/natal territory) from non-successive inheritors** | **Beta GLMM** | **n/a** | **Dispersion = 1.1541, p = 0.432** | **-103.2** |
| **Model 5** | **% of natal territory makeup (overlap/new territory) from non-successive inheritors** | **Beta GLMM** | **n/a** | **Dispersion = 1.0695, p = 0.584** | **233.9** |
